# Precariousness, Diabetes Control and Complications in French Guiana

**DOI:** 10.3389/fendo.2022.937156

**Published:** 2022-07-22

**Authors:** Samuel Linière, Mathieu Nacher, Kinan Drak Alsibai, Mayka Mergeayfabre, Nezha Hafsi, Aurelie Charpin, Caroline Misslin-Tritsch, Jean-François Carod, Jean Markens Aurelus, Bertrand De Toffol, André Ntoutoum, John Bukasa Kakamba, Magalie Demar, Jeannie Helene-Pelage, Antoine Adenis, Nadia Sabbah

**Affiliations:** ^1^ Department of General Medicine, Cayenne Hospital Center, Cayenne, French Guiana; ^2^ Clinical Investigation Center Antilles French Guiana (CIC INSERM 1424) Cayenne Hospital Center, Cayenne, French Guiana; ^3^ Department of Pathology and Center of Biological Resources (CRB Amazonie), Cayenne Hospital Center, Cayenne, French Guiana; ^4^ Department of Endocrinology and Metabolic Diseases, Cayenne Hospital Center, Cayenne, French Guiana; ^5^ Department of General Medicine, Ouest Guyanais Hospital Center, Saint-Laurent, French Guiana; ^6^ Laboratory of Biology, Ouest, Guyanais Hospital Center, Saint Laurent, French Guiana; ^7^ Department of Neurology, Cayenne Hospital Center, Cayenne, French Guiana; ^8^ Laboratory of Parasitology-Mycology (LHUPM), Cayenne Hospital Center, Cayenne, French Guiana; ^9^ EA3593, Amazon Ecosystems and Tropical Diseases, University of Guiana, Cayenne, French Guiana; ^10^ Department of General Medicine, University of the French West Indies and Guiana, Pointe-à-Pitre, Guadeloupe

**Keywords:** precariousness, diabetes, retinopathy, infections, stroke

## Abstract

**Aims:**

The social parameters of an individual impact the incidence of cardiovascular diseases. French Guiana, an overseas French territory with a lower standard of living than France, has a prevalence of diabetes mellitus that is twice that of mainland France. In this context we aimed to study the relation between precariousness, diabetes complications and glycemic control.

**Methods:**

A multicenter prospective cohort was initiated since May 2019. 1243 patients were included and their outcomes and history were compared between the precarious and non-precarious based on their EPICES score, a score that measures social isolation and precariousness.

**Results:**

73.3% of the sample was considered precarious. Retinopathy was significantly more frequent among the deprived. There were no significant differences for other macro or microvascular complications.There was a significant difference in Glycated Haemoglobin between the precarious and non-precarious groups (8.3% (67 mmol/l) vs 8.8% (73mmol/l)). After adjusting for potential confounders, precariousness was no longer associated with poor glycemic control; the independent factors significantly associated with poor glycemic control were: not being fluent in French, having creole or portugese as mother language, and not having any insurance.

**Conclusions:**

Precariousness is a risk factor for retinal complications in patients with diabetes mellitus in French Guiana. In this chronic disease, the universal healthcare system alleviates health inequalities for many, but not all, diabetic complications.Translation and cultural mediation may further reduce health inequalities in this multicultural territory where a substantial proportion of the population is not fluent in French.

## Introduction

The relationship between health and an individual’s social parameters has received renewed attention in recent years. In 2012, the World Health Organization’s regional council for Europe included in its “Health 2020” project the reduction of social inequalities in health as the main strategic and political objective (1). France is one of the Western European countries where social inequalities in health are the highest despite an improvement in the average standard of living (2).

It is now widely demonstrated that material and social deprivation in the general population is inversely related to health status (3). Indeed, in socially deprived populations, there is an increase in mortality (4). Thisis largely linked to an increase in the incidence of cancers (5)cardio vascular diseases and their risk factors (6).

Diabetes, which is increasing in incidence (7), is one of the major cardiovascular risk factors. Several publications describe the influence of social class on the mortality rate of diabetes (8) has a direct consequence of poor glycemic control and increased complications in vulnerable population (9).

To highlight these causal relationships, several indicators have been proposed to represent socio-economic status (10), the socio-occupational category (4), income (6) or the level of education (9). However, these inequality indicators do not assess the socio-economic status of an individual in all its dimensions (11). To define more precisely the link between the social parameters of an individual and his health, the concept of precariousness, defined in 1987 by Joseph Wresinski, seems to be more appropriate (12). This global dimension is similar to that used in the Anglo-Saxon literature by Peter Townsend in his terms of « material and social deprivation » (13).French Guiana is an overseas territory located at more than 7090Km from mainland France, it is one of the largest French territories with 90% of its 84000km2 covered by primary Amazonian forest. Remote populations are isolated because the road infrastructure is poor and means of transportation are rare. The population includes more than thirty ethnic groups and is culturally diverse. With the highest GDP per capita in Latin America it attracts numerous immigrants, notably from northern Brazil, Suriname, Guyana, and Haiti. Overall, 29% of the population is of foreign origin, and for adults it is nearly half of the population. The health system is that of France with universal health coverage, notably allowing the poorest to receive free health care, including undocumented immigrants.

All social indicators show that the standard of living in the overseas territories, and in particular in French Guiana, is lower than in mainland France (14).For example, the unemployment rate in French Guiana was 16.1% in 2020 compared to 7.8% in mainland France and the poverty level was 53% compared to 14% (14). At the same time, in these territories, there is a higher prevalence of cardiovascular disease (15) and diabetes (16).

Glycemic control of the patient with diabetes is key to prevent the risk of complications (17). However, few studies have simultaneously evaluated the link between precariousness, diabetes complications and glycemic control of the patient with diabetes and none has yet been conducted in the overseas French territories, which combine poor populations and a French health system. The main objective of the study was to evaluate the influence of precariousness on the complications of diabetes and on glycemic control.

## Materials and Methods

The CODIAM cohort (Cohort of Diabetes in French Amazonia) is a prospective cohort study conducted in French Guiana since May 2019 describing the epidemiological, clinical and biological aspects of different types of diabetes. It is a multicenter study conducted at the André Rosemon Hospital in Cayenne and the West Guianese Hospital in Saint-Laurent-du-Maroni, French Guiana, between May 2019 and June 2021. Given the number of different potential outcomes there was no predetermined sample size calculation. The aim was to include as many patients as possible to describe our population and to have the greatest possible power to identify trends.

Overall, 1243 participants with diabetes mellitus were included in the CODIAM cohort. Diabetes was defined as an increase in fasting plasma blood glucose greater than or equal to 126 mg/dL (or 7 mmol/L) on two occasions and/or a blood glucose level taken at any time of day greater than or equal to 200mg/dL (or 11 mmol/L) on two occasions.

### Inclusion Criteria

Patients over 18 years of age, with a confirmed diagnosis of diabetes and signed consent.

### Exclusion Criteria

Patients who were minors, under guardianship, did not sign consent, refused to participate, were deprived of liberty, in acute life-threatening situations, with gestational diabetes, or during pregnancy and post partum (less than 6 months) were excluded.

### Data Collection

Patients were included (first measure -V1) during consultations atthe Endocrinology Diabetology Nutrition department of Cayenne Hospital and the General Medicine department of Saint-Laurent-du-Maroni Hospital between May 2019 and June 2021. They were also recruited during hospitalizations in one of the medical or surgical departments of Cayenne hospital who requested an expertise in diabetology. Various data (social and demographic characteristics, medical and surgical history, examination data, and biological results, including basic diabetes monitoring parameters)were collected from included patients.

### Evaluation of Precariousness

The EPICES (Evaluation of Precariousness and Inequalities in Health in Health Examination Centers) score was initially developed from a 42-variable questionnaire selected from the major Townsend and Wresinski studies. These included traditional socioeconomic determinants such as education, income, and occupation, but also take into account questions about family structure and housing, social benefits, and leisure activities. After the creators of the score conducted factor analysis of the 42 variables and multiple regression of the “social precarity gradient,” they selected a subset of 11 binary questions (**Appendix 1**). These 11 questions are the ones that are used to compute the score.Each question has a regression coefficient, the score varies from 0 (no precariousness) to 100 (maximum precariousness).The definition of deprivation state is defined as a score ≥ 30.17, a threshold which was established in a large cohort study carried out by *Centre technique d’appui et de formation des centresd’examens de santé* (Technical Centre of Support and Training for Health Centres) (18, 19).

The micro and macro vascular complications of diabetes (retinopathy, nephropathy, neuropathy, coronary artery disease (angina pectoris pectoris or myocardial infarction), transient ischemic attack or stroke, obliterative arteriopathy of the lower limbs)werecompared between precarious and non precarious. Indicators of glycemic control and associated clinical data of the patient with diabetes were compared between precarious and non precarious individuals.Infections classically attributable to diabetes (cutaneous, urinary tract, pulmonary, osteoarticular) were also compared between precarious and non precarious groups.

### Statistical Analysis

Statistical analysis was performed using STATA software ®(STATA-CORP®, StataCorp LLC 4905 Lakeway Drive College Station, Texas 77845-4512 USA). A descriptive analysis of the study population and clinical data was performed. Quantitative data are expressed as mean and standard deviation and qualitative variables were expressed as frequencies and percentages. Comparisons of quantitative variables between precarious and non precarious patients were made using Student’s t-tests. The cross-tabulation of qualitative variables with the binary precariousness variable used the Chi-2 test to test for statistically significant differences. Glycated hemoglobin concentration was used as a dependent variable and other independent explanatory variables were included in a multiple linear regression model. The significance level was 5%.

### Regulatory and Ethical Aspects

All included patients were informed of the anonymous use of their data for the research. In accordance with the French Data Protection Act and the General Data Protection Regulation, the data processing was subject to a data protection impact analysis, an entry in the hospital’s data processing register and a declaration of compliance MR003. The protocol was approved by the Comité de Protection des Personnes Sud-Est de Clermont-Ferrand (Nos ref: 2020/CE 05). All patients provided written informed consent for participation and publication of anonymized study results.

## Results

### Sociodemographic, Clinical and Biological Characteristics of the Population

1243 patients were included in the CODIAM cohort during the study period, 1018 responded to the EPICES score questions. 751 patients, i.e. 73.3% of the sample, had an EPICES score > 30.17 and were thus considered as precarious (**Table 1**). In the precarious population, there were more women (57.3%) than men. The non-precarious population was significantly older (59.1 ± 12.2 vs 56.0 ± 12.7 years) than the precarious population (**Table 2**). The meanduration of diabetes was identical at 9.3 ± 9.3 years in non precarious population and 9.3 ± 9.1 years in precarious population. Regarding the clinical examination, the group of people in precarious situations were significantly shorter by 4 cm.

**Table 1:** Sociodemographic characteristics of the study population according to Precariousness.

	*N (n)*	*Non-precarious*	*Precarious*	*P*
*Social benefits*	1018			**<0.001**
*AME*	60	2 (0.8%)	58 (7.7%)	
*CMU*	173	27 (10.1%)	146 (19.4%)	
*ALD*	742	236 (88.4%)	506 (67.4%)	
*No *	43	2 (0.7%)	41 (5.5%)	
*Profession*	1018 (15)			**<0.001**
*No jobs ever*	255	27 (10.1%)	255 (34%)	
*Unemployed*	76	3 (1.1%)	73 (9.7%)	
*Retired*	221	78 (29.2%)	143 (19.0%)	
*In operation*	389	153 (57.3%)	236 (31.4%)	
*Other*	35	5 (1.9%)	30 (4.0%)	
*Schooling*	1018 (46)			**<0.001**
*No*	77	9 (3.4%)	68 (9.0%)	
*Primary*	186	17 (6.4%)	169 (22.5%)	
*Secondary*	236	44 (16.5%)	192 (25.6%)	
*Professional*	96	39 (14.6%)	57 (7.6%)	
*High School*	202	65 (24.3%)	137 (18.2%)	
*Bachelor*	39	18 (6.7%)	21 (2.8%)	
*Faculty*	110	41 (15.4%)	69 (9.2%)	
*Master*	26	19 (7.1%)	7 (1.0%)	
*Mother tongue*	1018			**<0.001**
*French*	152	79 (29.6%)	73 (9.7%)	
*Guyanese Creole*	253	85 (31.8%)	168 (22.4%)	
*Haitian Creole*	262	22 (8.2%)	240 (32.0%)	
*West Indian Creole*	62	27 (10.1%)	35 (4.7%)	
*Portuguese*	79	9 (3.4%)	79 (9.3%)	
*Spanish*	36	5 (1.9%)	31 (4.1%)	
*English*	55	8 (3.0%)	47 (6.3%)	
*Dutch*	11	1 (0.4%)	10 (1.3%)	
*Other*	108	31 (11.6%)	77 (10.2%)	
*Speaks French*	1003			**<0.001**
*Yes*	780	243 (91.3%)	537 (72.9%)	
*A little*	138	11 (4.1%)	127 (17.2%)	
*Not well*	46	7 (2.6%)	39 (5.3%)	
*No*	39	5 (1.9%)	34 (4.6%)	
*Writes/reads French*	995			**<0.001**
*Yes*	646	226 (86.3%)	420 (57.3%)	
*A little*	125	9 (3.4%)	116 (15.8%)	
*Not well*	79	8 (3.0%)	71 (9.7%)	
*No*	145	19 (7.2%)	126 (17.2%)	
*Marital status*	1018 (12)			
*Single*	400	52 (19.4%)	348 (46.3%)	
*Lives with a partner*	161	52 (19.5%)	109 (14.5%)	
*Civil Partnership*	10	3 (1.1%)	7 (0.9%)	
*Married*	308	133 (49.8%)	175 (23.3%)	
*Divorced/widowed*	127	26 (9.7%)	101 (13.4%)	

N: total data. n: missing data. AME: Aide Médicaled’Etat. CMU: complementary health insurance. ALD: long-term illness. values are represented by mean and standard deviation. P values computed with Chi2 tests except for variables followed by * for which t-tests were used.

**Table 2:** Medical characteristics of the study population according to precariousness.

	*N (mv)*	*Non-precarious*	*Precarious*	*P*
*Topics*	1018	267 (26.2%)	751 (73.3%)	**0.006**
*Men*	461	140 (52.4%)	321 (42.7%)	
*Women*	557	127 (47.6%)	430 (57.3%)	
*Type of diabetes*				
*Type 2*	911	244 (91.3%)	667 (88.9%)	
*Type 1*	56	16 (6.0%)	40 (5.3%)	
Age (years)*	911	59.1 (±12.2)	56.0 (±12.7)	**0.016**
Duration of diabetes (years)*	903	9.3 (±9.3)	9.3 (±9.1)	0.84
*Clinical examination*				
Size (cm)*	904	167.8 (±10.2)	163.8 (±10.1)	**<0.001**
Weight (kg)*	899	86.3 (±19.8)	84.1 (±19.5)	0.085
BMI (kg/m2)*	890	30.6 (±5.9)	31.4 (±6.9)	0.26
Temperature (°C)*	740	36.6 (±0.4)	36.7 (±0.4)	0.41
Systolic blood pressure (mmHg)*	901	140.7 (±17.4)	139.5 (±19.3)	0.21
Diastolic blood pressure (mmHg)*	900	79.5 (±12.5)	79.4 (±13.3)	0.90
*Biology*				
Clearance (mL/min)*	712	96.8 (±32.1)	98.9 (±32.5)	0.08
Microalbuminuria (mg/24h)*	659	70.5 (±200.5)	114.6 (±344.5)	0.72
*LDL (mmol/l)*	773	2.6 (±1.0)	2.6 (±1.0)	0.92
HBA1c (% [mmol/l])*	829	8.3[67] (±2.0)	8.8[73] (±2.1)	0.008
Creatinine (μmol/l)*	842	82.9 (±45.2)	84.7 (±63.7)	0.21
Lipoproteine(a) (mmol/L)*	627	412.2 (±455.6)	442.3 (±466.6)	0.25
CRP (mg/L)*	766	6.7 (±17.7)	7.5 (±15.9)	0.45
TSH (mIU/L)*	772	2.5 (±11.6)	1.7 (±1.2)	0.51
25 Vitamin D (ng/mL)*	788	27.7 (±10.3)	28.9 (±10.0)	0.08
*Background*				
*Renal insufficiency*	845 (19)	33 (14.5%)	82 (13.3%)	0.10
*High blood pressure*	844	155 (68.3%)	448 (72.6%)	0.28
*Depression*	839 (15)	16 (7.1%)	38 (6.2%)	0.90
*Blindness*	841 (4)	2 (0.9%)	15 (2.4%)	0.47
*Surgical*	1017 (14)	186 (69.9%)	472 (62.8%)	**0.024**
*Education*				
*Home Care Nurse*	1018 (91)	59 (22.1%)	316 (42.1%)	**<0.001**
*Therapeutic education*	1018 (112)	167 (62.5%)	545 (72.6%)	**0.009**
*Risk factors*				
*Alcohol*	1016 (12)			**<0.001**
* Occasional*	388	134 (50.4%)	254 (33.9%)	
* Chronic*	40	10 (3.8%)	30 (4.0%)	
*No*	576	121 (45.5%)	455 (60.8%)	
*Smoking*	1018 (13)			0.86
* Weaned*	94	25 (9.4%)	69 (9.2%)	
* Yes*	95	28 (10.5%)	67 (8.9%)	
*No*	816	210 (78.6)	606 (80.7%)	

N: total data. mv: missing data. CRP: C-reactive Protein TSH Thyroid Stimulating Hormone. IDM: myocardial infarction. values are represented by mean and standard deviation. P values computed with Chi2 tests except for variables followed by * for which t-tests were used.

Biologically, the precarious subjects had a 0.5% higher Glycated Haemoglobin level than the non precarious. The vitamin D level was higher in the precarious group, but this failed to reach statistical significance. No significant difference was found for the rest of the variables.

Unsurprisingly, patients in the precarious group were significantly more likely to benefit from the PUMA, AME (**Table 1**) (guarantees any person working or residing in France in a stable and regular manner: Uninterrupted residence in France for more than 3 months a right to coverage of his or her health expenses on a personal basis and continuously throughout life) and/or CMUc health insurance regimens designed for the poor.

The population without a professional activity was significantly more important in the precarious group (**Table 1**), while the population with aprofessional activity was more important in the non precarious group. The level of education was inversely proportional to precariousness. The precarious population had significantly more difficulty speaking, reading and writing French and less than 10% considered French as their mother tongue.

No significant differences were shown in chronic alcohol use and active smoking (**Table 2**).

Finally, the precarious group was significantly more likely to benefit from a home care nurse and to participate in therapeutic education sessions.

### Complications 

Retinopathy and infections were significantly greater in the precarious group than in the non precarious group. No significant difference was shown fornephropathy, but there was an increase in microalbuminuria in the precarious group without alteration of renal function (**Table 3**).

**Table 3:** Complications of the patient with diabetes according to precariousness.

	*N*	*mv*	*Non-precarious*	*Precarious*	*p*
*Diabetic nephropathy*	1018	79	57 (21.3%)	184 (24.5%)	0.39
*Diabetic retinopathy*	1018	162	28 (10.5%)	101 (13.4%)	**0.03**
*Diabetic foot*	1018	46	14 (3.0%)	40 (2.1%)	0.29
*Diabetic neuropathy*	1018	58	27 (10.1%)	75 (10.0%)	0.77
*Diabetic arteriopathy*	1016	128	20 (7.5%)	50 (6.7%)	0.67
*Angina pectoris*	619	14	6 (3.6%)	23 (5.1%)	0.58
*Infection*	938	98	26(10.4%)	67(9.7%)	0.74
*Stroke*	920	30	58 (22.6%)	144 (21.7%)	0.60
*Transient ischemic attack*	619	14	10 (5.9%)	18 (4.0%)	0.33
*Hypoglycemia*	619	14	20 (11.9%)	52 (11.5%)	0.88
*Hyperosmolar coma*	619	23	7 (4.2%)	19 (4.2%)	0.93
*Ketoacidosis*	617	19	10 (1.8%)	38 (3.5%)	0.33

N, total data; mv, missing values.

### Multiple Linear Regression Model of Glycated Hemoglobin Concentration 

After adjusting for potential confounders, precariousness was no longer associated with glycated hemoglobin concentration. The variables that remained independently associated with a significant increase in the concentration of glycated hemoglobin were: being poorly fluent in French (+1.2% than fluent), having as a mother language Haitian creole (+0.6% relative to French), Guianese creole (+0.6% relative to French), Antilles creole (+0.7% relative to French) and portugese (+0.9% relative to French), and not having any health insurance (+1.5% relative to Full ALD insurance for diabetes) (**Table 4**).

**Table 4:** Multiple linear regression model of glycated hemoglobin concentration and demographic and socioeconomic independent variables. .

	Coefficient	[95% ConfidenceInterval]	P
**Maternal language**			
*French*	Reference		
*Guianese Creole*	0.57	0.07;1.00	**0.025**
*Haitian Creole*	0.64	-0.02;1.08	0.057
*Portugese*	0.87	0.16;1.54	**0.016**
*Spanish*	0.20	-0.68;1.07	0.659
*English*	0.32	-0.44;1.08	0.403
*Dutch*	1.18	-0.27;2.64	0.111
*Antilles Creole*	0.79	0.13;1.46	**0.019**
*Other*	-0.01	-0.65;0.63	0.970
**Precariousness (present/absent)**	0.03	-0.33;0.38	0.875
**Age (years)**	-0.03	-0.04;-0.01	**0.000**
**Diabetes type**			
*Type 1*	Reference		
*Type 2*	-0.33	-0.98;0.32	0.324
*Type Slow*	-0.18	-1.50;1.14	0.791
*Type Mody*	-0.02	-4.22;4.18	0.993
*Other type*	0.78	-0.18;1.73	0.113
**Health insurrance**			
*None*	1.34	0.56;2.11	**0.001**
*AME State Insurrance*	-0.17	-0.87;0.53	0.632
*CMU Welfare*	-0.01	-0.40;0.39	0.974
*ALD Insurance (Fully insurred for diabetes)*	Reference		
**Profession**			
*None*	-0.15	-0.98;0.68	0.727
*Unemployed*	-0.11	-1.03;0.81	0.811
*Never worked*	1.21	-0.02;2.45	0.054
*Retired*	-0.27	-1.15;0.61	0.549
*Active*	0.25	-0.55;1.05	0.542
*Other jobs*	Reference		
*Unknown*	0.18	-1.37;1.74	0.816
**Education**			
*None*	-0.50	-1.32;0.31	0.222
*Primary*	0.15	-0.48;0.77	0.638
*Secondary*	-0.35	-0.90;0.20	0.217
*Vocational*	-0.91	-1.54;-0.27	**0.005**
*High school*	-0.34	-0.88;0.20	0.220
*College*	Reference		
*Bachelor*	-0.36	-1.16;0.43	0.373
*Masters*	-0.26	-1.24;0.71	0.594
*Unknown*	-0.05	-0.89;0.79	0.908
**Speaks**			
*No French*	-0.16	-1.12;0.81	0.753
*Poorly fluent*	1.19	0.32;2.07	**0.008**
*A little fluent*	0.07	-0.49;0.62	0.810
*Fluent*	Reference		
**Writing**			
*No French*	-0.32	-1.00;0.36	0.360
*Poor French*	-0.13	-0.77;0.52	0.701
*A little French*	0.11	-0.44;0.65	0.704
*Writes French*	Reference		
**Nurse**			
*No nurse assistance*	Reference		
*Unknown nurse*	0.37	-0.17;0.90	0.178
*Nurse assistance*	0.16	-0.16;0.47	0.330
**Alcohol**			
*Non drinking*	Reference		
*Unknown*	-0.68	-2.38;1.02	0.432
*Drink alcohol*	-0.13	-0.44;0.17	0.398
*Misuses alcohol*	-0.82	-1.54;-0.10	**0.025**
**Therapeutic education**			
*No therapeutic education*	Reference		
*Unknown therapeutic education*	-0.35	-0.89;0.19	0.208
*Therapeutic education*	-0.02	-0.40 ;0.34	0.884

## Discussion

This work showed the association of precariousness with glycemic imbalance and retinal complications in patients with diabetes mellitus. However, apart from these significant results, the negative results may be even more striking: indeed, counter to our initial expectations, for most macro and microvascular complications there were no significant differences between precarious and non precarious patients. Furthermore, after adjusting for potential confounders precariousness was no longer associated with glycated hemoglobin concentration; the variables that remained independently associated with a significant increase in the concentration of glycated hemoglobin were: being poorly fluent in French, not having any health insurance, having creole or portugese as a mother language. Not understanding what the health professionals say and not being insurred are known obstacles to glycemic control: Management of diabetes requires interactive communication between the patient and the physician and shared decision making (20). Indeed, greater efficiency in health behaviors, health literacy allow for better glycemic control (21, 22). The mother language variable, which was adjusted for insurance and fluency, suggests other complementary explanations: cultural or genetic differences that may also influence glycemic control. Studies have indeed shown that ethnic minorities often have poor glycemic control (23). These differences are thought to be related to different biological functions related to insulin resistance, hyperinsulinemia, beta cell function and fat metabolism (24). It may have seemed surprising to find, after adjustments, no relation of having a home nurse or therapeutic education with glycemic control. However, the relation between glycated hemoglobin and these 2 variables actually goes both ways: although the interventions improve glycemic control, doctors prescribe a home nurse and more therapeutic education when glycemic control is poor.

Overall, the results suggest that for this chronic disease, once it is diagnosed, the universal health system eventually rectifies social inequalities in health in the long run leading to few differences between the precarious and non precarious, Hence, precarious patients were significantly more likely to have a home nurse than the non precarious (42.1% vs 22.1%) and more therapeutic education (81% vs 72%) to try to compensate for patient difficulties.

Given the scarcity of ophtalmologists in French Guiana, the increased proportion of patients with diabetic retinopathy among the precarious possibly reflects the particular difficulty to accessthe specialty for precarious populations (25). In French Guiana, only 19% of patients with diabetes had their eyes checked at least once every two years (26).

We did not find any significant difference between the two groups concerning diabetic nephropathy, unlike some authors (27) but similarly to others (28). We did not find any significant difference in angina pectoris or stroke, which differs from the literature (29, 30), probably because of the small number of events but perhaps also because the imperfect but universal healthcare system manages to provide primary care to the disfavored groups thus erasing the risk difference with the non-precarious.

In our population of patients with diabetes, a massive 73.3% of subjects were precarious. Precarious patients were older and more likely to be female than non precarious patients, presumably because with time and age, persons acquire greater social capital and resources whereas for single women, caring for children is costly. Comparative studies carried out in mainland France on a diabetic population using the same score to define precariousness show a prevalence ranging from 6.9% to 46% (19). In French Guiana, the prevalence of precariousness in a population witha stroke was 69% (31). In the United States, inequality among Afro-American, Hispanic or Asian ethnic minorities have been highlighted (32). The underlying mechanisms arein part explained by different lifestyles, particularly in terms of culture, understanding of the disease, and access to care, which can be very variable between health systems (33).

The EPICES score is regularly used in epidemiological studies dealing with the relationship between an individual’s socioeconomic factors and health status (34). It has been compared with the Anglo-Saxon indicators of Townsend and Carstairs and has shown equivalent results for the measurement of material and social deprivation (35). One study highlighted its capacity to measure precariousness in a multidimensional way and its robustness in the detection of precarious patients (36). Furthermore, a recent study comparing French Guiana to mainland France (37) showed no major difference between the two for the detection of precariousness and the EPICES score.

The strength of our study lies is that it allowed us to obtain a diverse sample of the French Guianese population and thus to limit selection bias. The weaknesses of the study lie mainly in the short duration of the follow-up of the cohort and future work will focus on the follow-up of the cohort over 24 months.

Furthermore, although the reduction of health inequalities have been a priority in many countries, to our knowledge, our work is one of few that has compared the occurrence of clinical events in a population with diabetes between the precarious and non precarious.

In conclusion, although there was a link between precariousness and the risk of retinopathy in precarious patients, this work highlights the fact there were no other significant differences between precarious and non precarious patients regarding micro or macrovascular complications of diabetes. Our health system may have difficulties in reaching some populations, however for chronic diseases such as diabetes, once patients are in the health care system, it seems to be fairly effective in erasing differences between the precarious and the non precarious. One of the weakest points of the French health system operating in the multicultural and multilingual context of French Guiana is the lack of translators and cultural mediators to cover the quasi-constant needs. The present results suggest this may be an area where gains could be made. There is still room for expanding the reach of the universal health system and mobile teams are being set up to go to isolated areas, therapeutic education modules are being strengthened in the departments with cultural and linguistic adaptation, and health mediators to improve glycemic control.

## Data Availability Statement

The original contributions presented in the study are included in the article/**Supplementary Material**. Further inquiries can be directed to the corresponding author.

## Ethics Statement

The studies involving human participants were reviewed and approved by ref: 2020/CE 05. The patients/participants provided their written informed consent to participate in this study.

## Author Contributions

Conceptualization: SL, MN, MM, AA, NS. Data curation: SL, MN, KD, MM, NH, AC, JA, AN, JK, AA, NS. Formal analysis: SL, MN, NS. Investigation: SL, MN, NH, MM, AC, CM-T, J-FC, JA, AA, NS. Resources: SL, MN, KD, MM, NH, AC, CMT, J-FC, JA, MD, KD AN, AA, NS. Supervision: MN, AA, NS. Validation: SL, MM, NH, MM, AC, CM-T, J-FC, JA, BD, KD JH-P, AN, JK, MD, AA, NS. Visualization: SL, MM, NH, KD, MM, AC, CM-T, J-FC, JA, BD, MD, JH-P, AN, AA, NS. Manuscript writing: SL, MN, KD, AA, NS. All authors contributed to the article and approved the submitted version.

## Conflict of Interest

The authors declare that the research was conducted in the absence of any commercial or financial relationships that could be construed as a potential conflict of interest.

## Publisher’s Note

All claims expressed in this article are solely those of the authors and do not necessarily represent those of their affiliated organizations, or those of the publisher, the editors and the reviewers. Any product that may be evaluated in this article, or claim that may be made by its manufacturer, is not guaranteed or endorsed by the publisher.

## Abbreviations

CODIAM, Cohort of Diabetes in French Amazonia; EPICES, Evaluation of Precariousness and Inequalities in Health in Health Examination Centers; AME, state medical aid; ALD, Long-term care insurance; PUMA, universal health protection; CMU-c, complementary universal health coverage; HbA1c, Glycated Haemoglobin.
