# Supplementary material for: Precariousness, Diabetes Control and Complications in French Guiana
Source: Front Endocrinol (Lausanne). 2022 Jul 22;13:937156. doi: 10.3389/fendo.2022.937156 (PMC9355552; doi:10.3389/fendo.2022.937156)
Supplement: Supplementary file 1 [file Table_1.pdf]

Appendix 1: The EPICES score

Number	questions	yes	no
1	Do you ever meet with a social worker?		
2	Do you have supplementary health insurance?		
3	Do you live in a couple?		
4	Do you own your home?		
5	Are there periods in the month when you have real financial difficulties to meet your needs (food, rent, electricity...)?		
6	Have you played any sports in the past 12 months?		
7	Have you been to a show in the last 12 months?		
8	Have you gone on vacation in the last 12 months?		
9	In the past 6 months, have you had contact with family members other than your parents or children?		
10	In case of difficulties, are there people around you who you can count on to put you up for a few days in case of need?		
11	In case of difficulties, are there people around you who you can count on for material help?		
	constant		
